# The Royal Infirmary, Edinburgh

**Published:** 1916-01-15

**Authors:** 


### PATRIOTISM IN BRITISH HOSPITALS.*

### III. The Royal Infirmary, Edinburgh.

There can be no fitter comment on the rôle played by the students and graduates of the Edinburgh School of Medicine attached to the Royal Infirmary, Edinburgh, than the message received from the Chancellor of the University, the Right Honourable A. J. Balfour, on Graduation Day last July. "From our University there have gone forth to every corner of the world uncounted multitudes skilled in letters, in science, and in all the arts of healing. I am proud to think that when their country calls and the freedom of mankind is in the balance, the Edinburgh students of to-day have proved themselves not less admirable in the stress of war than were their predecessors in the calm of peace."

The multitudes are still uncounted. But a gallant attempt has been made to count them. The list of names supplied to us fills seventy-six closely printed pages, and includes all the graduates and undergraduates of Edinburgh University known to be doing war work. Their degree or status is appended with the office they now hold, and thus with the aid of the admirable statistical summary drawn up by Mr. James Walker, C.A., it is possible to disentangle from the total those who were members of the medical school. This gathering together of uncounted multitudes of past and under graduates in the defence of country and homeland will undoubtedly bear abundant and golden fruit after the war. We can only regret that our space is too limited to enable us to set out in the case of Edinburgh and every other University and School the entire lists of names.

### Edinburgh Infirmary Men in the War.

Taking the graduates first, it appears that 124 hold commands as surgeons in the Navy, and one is in the ranks. There are no fewer than 1,285 Edinburgh Infirmary men holding commissions in the Royal Army Medical Corps and the Territorial Force. Nine medical graduates are serving in the ranks and 91 are auxiliaries. Of the *alumni*, as distinguished from graduates proper, one is a naval surgeon, four have commissions in the medical services of the Army, three are in the ranks, and eight are auxiliaries.

There are 37 undergraduates who hold commissions as Naval Surgeons, 31 who hold commissions in the R.A.M.C., 88 in the ranks, and 18 auxiliary.

Great sacrifices have been made at the Royal Infirmary for the national cause. A total of 29 members of the teaching staff of the medical school have left to take up war work, and these include professors, tutors, and lecturers on clinical surgery and medicine, on chemistry, anatomy, materia medica, and various special subjects. For the most part they have taken commissions in the R.A.M.C. and are serving in military hospitals at home or abroad. Two are surgeons in the Navy.

### Pro Patria.

Among those who have laid down their lives for their country are ten Edinburgh medical students, several of whom were serving as combatants. Mr. A. E. Turnbull, M.B., surgeon R.N. Reserve, was drowned in action on H.M.S. *Cressy*, Dr. Fernand Louis de Verteuil on H.M.S. *Good Hope*, Surgeon J. H. D. Watson on H.M.S. *Hawke*, and Surgeon Hugh J. Hopps on H.M.S. *Aboukir*. Fifteen other graduates of the medical school were killed in action or died of wounds. Among those wounded are four medical students and 29 former graduates. Dr. H. C. Hinwood, M.B., R.F.A., and Dr. James Lindsay, of the West African Medical Staff, are prisoners of war.

### List of Honours.

Surgeon-General W. G. Macpherson, M.B., has been made Companion of the Bath. Surgeon-Probationer J. A. Stirling has obtained the Military Cross. Dr. Hamed Mahmud, a surgeon with the French Red Cross, has been recommended for the French Legion of Honour. Forty-seven graduates of the Edinburgh School of Medicine have been mentioned in dispatches for gallantry in action or distinguished services to the wounded. Of these two were killed, Captain Glanvill, R.A.M.C., attached to Royal Scots Greys, killed in action November 2, 1914, and Lieutenant J. L. Huggan, M.B., killed in action September 16, 1914.

Recent awards of honours include the Distinguished Service Order for Captain Whiteford J. E. Bell, M.B., R.A.M.C.; Major R. W. Knox, M.B., I.M.S.; Captain A. J. A. Menzies, M.B., R.A.M.C.; and Captain S. Alwyn Smith, M.B., Canadian R.A.M.C. The Military Cross has been bestowed on Lieutenant D. C. Alexander, M.B., R.A.M.C.; Lieutenant B. Score Browne, M.B., R.A.M.C.; and Captain G. Rankine, M.B., R.A.M.C. Among those recently mentioned in dispatches is Captain J. J. H. Nesson, M.D., I.M.S.

### Edinburgh School of Medicine for Women.

A high tribute has recently been paid by Dr. Wallace Williamson to the work of the Scottish women's hospitals, on the occasion of a recent sale in Edinburgh in their aid. In pointing out that this was a time of great opportunity for women both for service and sacrifice, he spoke warmly of the work of the Scottish women's hospitals and of the great work that these women doctors were doing both in France and Serbia. The tale of patriotism shown by members of the great medical school in Edinburgh would not be complete without reference to the fine services rendered by the women doctors.

*Articles Nos. I. and II. of this series appeared in our issues of November 13 and December 11, 1915.

